# Nanoscale “Quantum” Islands on Metal Substrates: Microscopy Studies and Electronic Structure Analyses

**DOI:** 10.3390/ma3073965

**Published:** 2010-07-09

**Authors:** Yong Han, Barış Ünal, Dapeng Jing, Patricia A. Thiel, James W. Evans, Da-Jiang Liu

**Affiliations:** 1 Institute of Physical Research & Technology, Iowa State University, Ames, IA 50011, USA; 2 Ames Laboratory, Departments of Materials Science & Engineering and Chemistry, Iowa State University, Ames, IA 50011, USA; E-Mails: barisunaltr@gmail.com (B.U.); dpjing@iastate.edu (D.J.); thiel@ameslab.gov (P.A.T.); 3 Ames Laboratory, Departments of Physics & Astronomy and Mathematics, Iowa State University, Ames, IA 50011, USA; 4 Ames Laboratory, Iowa State University, Ames, IA 50011, USA; E-Mail: dajiang@fi.ameslab.gov (D-J.L.)

**Keywords:** quantum size effect, metal nanofilms, quantum islands, DFT calculations, STM, Ag/Fe, Cu/Fe/Cu, Pb/Cu, Ag/NiAl, films on quasicrystals

## Abstract

Confinement of electrons can occur in metal islands or in continuous films grown heteroepitaxially upon a substrate of a different metal or on a metallic alloy. Associated quantum size effects (QSE) can produce a significant height-dependence of the surface free energy for nanoscale thicknesses of up to 10–20 layers. This may suffice to induce height selection during film growth. Scanning STM analysis has revealed remarkable flat-topped or mesa-like island and film morphologies in various systems. We discuss in detail observations of QSE and associated film growth behavior for Pb/Cu(111), Ag/Fe(100), and Cu/fcc-Fe/Cu(100) [A/B or A/B/A], and for Ag/NiAl(110) with brief comments offered for Fe/Cu_3_Au(001) [A/BC binary alloys]. We also describe these issues for Ag/5-fold i-Al-Pd-Mn and Bi/5-fold i-Al-Cu-Fe [A/BCD ternary icosohedral quasicrystals]. Electronic structure theory analysis, either at the level of simple free electron gas models or more sophisticated Density Functional Theory calculations, can provide insight into the QSE-mediated thermodynamic driving force underlying height selection.

## 1. Introduction

Scanning probe microscopy studies, especially scanning tunneling microscopy (STM) and atomic force microscopy (AFM) investigations, have provided an exquisitely detailed picture of the evolving morphology of growing films for both homoepitaxial and heteroepitaxial systems [1,2,3,4,5,6,7,8,9,10]. Electron microscopies have also provided valuable insights. Particularly prominent have been studies for metal-on-metal, semiconductor-on-semiconductor, and metal-on-semiconductor systems.

For “simple” metal homoepitaxial systems (A on A), fractal and dendritic 2-dimensional (2D) islands have been observed by STM during submonolayer deposition. Also dramatic wedding-cake like mounds (multilayer stacks of 2D islands resembling geographical morphologies of the south-west US canyon lands) have been observed during multilayer deposition [4,5]. These complex morphologies, occurring in systems with simple equilibrium states, reflect the feature that deposition drives the system far-from-equilibrium. For metal heteroepitaxy (A on B), fractal or dendritic island have been observed similar to those in homoepitaxy [1,2]. However, intermixing of A and B can produce different types of complex alloy overlayer structures [3]. A similar result was obtained by co-deposition (A + B on C) resulting in striped, droplet, or other structures [11,12]. For semiconductor heteroepitaxial systems, STM and AFM studies have revealed often elongated submonolayer islands (due to substrate anisotropy associated with reconstruction) and sometimes multilayer mounds similar to metal systems [6,13]. For semiconductor heteroepitaxy, a key focus has been on the strain-induced formation of well-separated 3D islands or “quantum dots” [7,8].

There have been extensive studies of metal-on-semiconductor heteroepitaxy going back decades [14]. However, a remarkable discovery was made in 1996 in STM studies by Shih and coworkers of Ag deposition on GaAs(110). Under suitable deposition conditions, they observed the development of perfectly flat films of a “magic” thickness [15]. If the total coverage was below this thickness, then the coverage deficit was accommodated by including within the flat film a number of pits extending down to the substrate. These Ag/GaAs(110) films are in fact metastable, and STM studies have also explored the morphological evolution during subsequent equilibration [16]. There has also been immense interest in the Pb/Si(111) system which exhibits a bilayer oscillatory stability, and also the formation of metastable mesa-like islands under suitable low temperature (*T*) deposition conditions, those of a “magic” height of 7 layers being particularly stable [9,10]. While the discovery of this behavior utilized low-energy electron diffraction (LEED) studies [17], many subsequent STM studies have characterized these mesa-like morphologies, as well as their formation or perturbation [9,10,18,19]. Their origin lies in quantum size effects (QSE) associated with electron confinement in the metal nanostructures, detailed behavior and height selection also being influenced by charge spilling and Friedel oscillation effects [20]. These features have now been observed in many other metal-on-semiconductor systems.

In fact, the formation of dramatic flat-topped film morphologies reflecting height-selection guided by QSE has also been observed in metal-on-metal heterostructures. These observations are the focus of the current contribution. A classic example is Pb on Cu(111) where there exist extensive experimental STM studies [21,22], and angle-resolved photoemission (ARPES) studies to reveal and analyze quantum well states (QWS) [23] underlying the QSE. There have also been theoretical analyses by several groups including semi-empirical modeling [24,25] and DFT studies [26,27]. Another classic system is Ag on Fe(100) where photoemission spectroscopy studies have been performed by Chiang and coworkers to assess electron confinement and associated QWS [28,29,30]. Subsequent LEEM studies by Altman and coworkers [31,32] characterized morphological evolution towards preferred heights. A particular significant and appealing feature of the Ag/Fe(100) system is the very good lateral lattice-match between the Ag(100) overlayer and the substrate. This results in a simple well-defined interface with Ag at four-fold hollow sites on the substrate. In contrast to the other systems described above, the unambiguous well-defined interface and coherent fcc(100) epitaxial structure of the overlayer for Ag/Fe(100) has allowed high-level DFT analyses of energetics for the *supported* film by Chou and coworkers [33]. The results were consistent with experimental observations. Since these earlier seminal investigations, other STM studies have indicated height selection in several other metal-on-metal heteroepitxial systems and QSE has been proposed as the underlying cause. Some of these examples, as well as Pb/Cu(111) and Ag/Fe(100), are reviewed here.

Naturally, in metal-on-semiconductor (or metal-on-insulator) systems, there can be strong confinement of electrons in the metal overlayer or nanostructure. For metal-on-metal systems, the electrons in the overlayer can also be confined if the substrate presents a relative band gap in the direction perpendicular to the surface. In this case, electrons cannot propagate into the substrate and are completely reflected at the film-substrate interface [34,35]. Confinement can even be produced by a relative ‘‘symmetry gap’’ [35]. This occurs, for example, if the film valence electrons close to the Fermi level have *sp* character and the substrate *sp* partial density of states has a gap at the Fermi level.

It should be noted that the mere occurrence of bilayer or multilayer islands in heteroepitaxial systems does not necessarily imply QSE-controlled growth morphologies. Heteroepitaxy generally produces 3D islands when the surface energy of the overlayer material is less than half the adhesion energy of the overlayer to the substrate [36]. The latter can depend on film thickness. This behavior can take the form of Volmer-Weber (VW) growth of 3D islands directly on the substrate, or Stranski-Krastanov (SK) growth where a wetting layer is first formed. This thermodynamic driving force when combined with kinetic limitations of higher layer nucleation could also produce, e.g., bilayer islands. It should also be noted that the surface energy will invariably depend on film thickness in heteroepitaxy. However, one generally expects a monotonic variation of surface energy and other properties in the absence of QSE, whereas QSE can induce more complex, e.g., oscillatory behavior. Another issue is whether the observed height-selected multilayer islands constitute the stable equilibrium state or just a metastable local minimum. Certainly, metal-on-semiconductor quantum islands are often metastable and in fact can be formed only by a suitable low-temperature deposition protocol to avoid the system evolving directly to the thermodynamic state (often 3D SK islands) [9].

An ability to understand and control the formation of height-selected flat-topped islands and films would have significant value for nanotechnological applications. If the size of any nanostructure is comparable to its corresponding electron Fermi wavelength, numerous physical and chemical properties may exhibit strong size dependence due to effects of quantum confinement of electrons [34,35,37,38,39,40,41,42,43,44]. For metal films of nanoscale thickness of up to about 10–20 atomic layers, many physical quantities vary or even oscillate as a function of film thickness. These quantities include thermodynamic stability [22,29,45,46], electrical resistivity [47], superconducting critical temperature [48], the perpendicular upper critical field [49], Hall coefficient [50], surface adhesion [51], thermal-expansion coefficient [52], surface free energy [53,54], surface diffusion barriers [54,55,56], surface adsorption energy [54,57], work function [44,58,59], electron density [44], *etc*. Here, we succinctly describe metal films of nanoscale thickness as “nanoislands”. If their morphology or properties are impacted by QSE, we further describe them as “quantum nanoislands”.

In Section 2, we provide some background on relevant electronic structure issues for confined electrons and associated QSE. Then, we review behavior for Pb/Cu(111) in Section 3, Ag/Fe(100) in Section 4, Cu/fcc-Fe/Cu(100) in Section 5, Ag/NiAl(110) in Section 6, Fe/Cu_3_Au(0001) in Section 7, and Ag/5-f i-Al-Pd-Mn and Bi/5-f i-Al-Cu-Fe in Section 8. Conclusions are provided in Section 9.

## 2. Background: Electronic Structure and QSE

**Figure 1:**
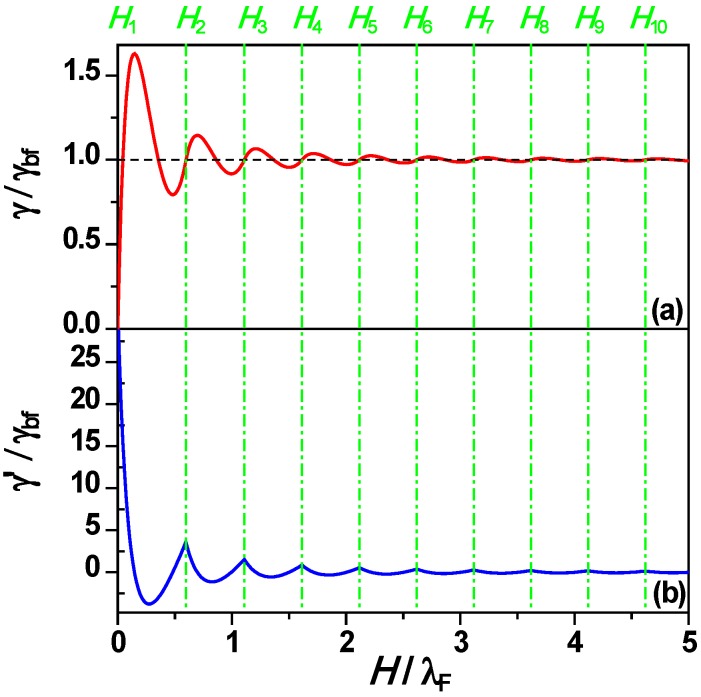
(A) Surface free energy γ, and (b) first derivative γ’ *versus* nanofilm thickness *H* from Equation (2). Dashed black horizontal line in (a) corresponds to bulk film surface free energy γ_bf_ and green dash-dotted vertical lines represent *H_n_* from Equation (1). Adapted from [67] (Copyright American Physical Society 2009).

A simplistic but useful assessment of QSE in metal nanofilms can be provided by a non-interacting electron-gas model (EGM) noting the nearly free-electron property of metals [60,61,62,63,64,65,66,67]. A further severe simplifying assumption of complete confinement allows description of the metal nanofilm as a free electron slab using a square-well potential well with an infinite-height barrier. We use a canonical ensemble approach to account for charge spilling by suitably shifting the location of the barrier from the physical film edge. Detailed analysis [67] of this EGM shows that Fermi energy level ε_f_ is oscillatory as a function of slab thickness *H*, and a series of cusps appear on the curve of ε_f_ *versus* *H*. These cusps correspond to the crossings of quantum well state (QWS) subbands and Fermi energy level. The positions of the cusps satisfy [67]

(1) $$H_{n}=\frac{\lambda_{\text{F}}}{2}\left(n-\frac{3}{4}-\frac{1}{12n}-\frac{1}{24n^{2}}-\cdots \right)$$

where *n* = 1, 2, 3, …, and λ_F_ is the Fermi wavelength for a bulk metal in the standard free-electron-gas model, *i.e.*, the Drude-Sommerfeld model. Equation (1) actually constitutes an almost linear variation of *H_n_* *versus* *n* with the slope of λ_F_/2, and thus the oscillation period of the curve of ε_f_ *versus* *H* is approximately equal to λ_F_/2. This behavior imposes an oscillatory form as a function of thickness *H* with the period of ~ λ_F_/2 on other key properties, most significantly for the current contribution on the surface free energy, γ(*H*). An analytic expression is available for this free energy of the form [67]

(2) $$\gamma (H)=10\gamma_{\text{bf}}\left[\frac{16}{9n}{\left(\frac{H}{\lambda_{\text{F}}}\right)}^{2}+\frac{H(n+1)\;(2n+1)}{9\;\lambda_{\text{F}}\;{\left(\frac{H}{\lambda_{\text{F}}}+\frac{3}{8}\right)}^{2}}-\frac{n\;(n+1)\;(2n+1)\;(8n^{2}+3n-11)}{2880\;{\left(\frac{H}{\lambda_{\text{F}}}+\frac{3}{8}\right)}^{4}}-\frac{8H}{5\;\lambda_{\text{F}}}\right]$$

for *H_n_* ≤ *H* ≤ *H_n+_*_1_, (2), where γ(*H*) → γ_bf_ (the bulk film surface energy), as *H* → ∞. Figure 1a and Figure 1b show γ and its first derivative γ’ *versus* nanofilm thickness *H* from Equation (2). The green dash-dotted vertical lines indicate that the crossings of subbands and Fermi energy level correspond to a series of inflection points on the curve of γ *versus* *H*. This leads to a phase shift of ~ λ_F_/4 between the maxima of the γ curve *versus* *H* and those of other electronic properties (e.g., chemical potential, work function, *etc*.) *versus* *H* [66,67].

In this EGM, the thickness *H* can be regarded as continuous. However, for a real metal nanofilm, the thickness only takes the discrete values, *Ld*, where *L* = 1, 2, 3,.... is the number of complete atomic layers or monolayers (ML) and *d* is the interlayer spacing. Consequently, one just selects discrete values from the corresponding continuous form for γ(*H*) *etc*. To facilitate analysis of the oscillation period for a real nanofilm with the discrete thickness *L*, a simple useful rule can be readily obtained from Equation (1). If the interlayer spacing *d* is commensurate with an integer multiple of λ_F_/2 [44], *i.e.*, if

(3) $$jd\approx m\frac{\lambda_{\text{F}}}{2},$$

where both *j* > 1 and *m* are the smallest possible positive integers with no common factor, then the metal film will display oscillatory behavior with a period of *jd*. However, for a specific metal film, generally speaking, *jd* (including *j* = 1) is never exactly equal to *m*λ_F_/2, and this results in a more complicated oscillatory pattern. In the special case where *m*λ_F_/(2*d*) is quite close but not exactly equal to the integer *j* then an oscillatory pattern with a period of Λ*d* occurs, where [54,67]

(4) $$\Lambda =\frac{\lambda_{\text{F}}/2}{\left|m\lambda_{\text{F}}/2-jd\right|}.$$

For example, for Pb(111) or Ag(110) nanofilms with a prominent bilayer oscillation, there is an additional envelope with the period of Λ*d* looks like a “beating” pattern, as described in detail below.

The above analysis does not include a realistic treatment of the interface between the film and the substrate. Aside from DFT analyses described below, there do exist some semi-empirical analyses intended to provide insight into the strength of electron confinement and related features of the associated QWS [24,25]. However, it should be emphasized that the oscillatory behavior described above is robust, the detailed nature of the interface generally just introducing a “phase shift” relative to the simple EGM results described above [54,67].

A much higher level of analysis of QSE can be provided by DFT analysis, readily for freestanding slabs or “films”, but also for supported epitaxial films if the interface structure is known. Typically such analyses are performed using periodic slabs with a plane-wave basis exploiting codes such as VASP [68]. In our analysis, we use the Perdew-Burke-Ernzerhof form of the generalized gradient approximation [69], and electron-ion interactions are described by the projector augmented-wave approach [70]. The primary quantity of interest is again the surface free energy which can be used to judge the stability of a film, lower energies corresponding to more stable films. For a freestanding metal slab or film, the surface free energy as a function of thickness *L* is calculated as

(5) $$\gamma_{L}=\frac{E_{L}-N_{L}E_{\text{c}}}{2A}$$

where *E_L_* is the total energy of the system for the supercell, *N_L_* is the total number of atoms in the supercell, *E*_c_ is the cohesive energy per atom for the bulk metal, and *A* is the area of the bottom or top surface of the supercell. Thus, by calculating *E_L_* and *E*_c_, the surface free energy γ*_L_* can be obtained. For a supported metal film, the surface free energy cannot be simply calculated from Equation (5) because of complications related to the substrate, and instead we consider [54]

(6) $$\alpha_{L}\equiv \gamma_{\text{t}}+\gamma_{\text{b}}+\gamma_{\text{i}}+\gamma_{\text{t,0}}+\gamma_{\text{b,0}}=\frac{E_{L}-E_{0}-N_{L}E_{\text{c}}}{A}$$

corresponding to the *relative* surface free energy of the film. Now, *E_L_* is the total energy of the system including the substrate for the supercell, and *N_L_* is the total atom number in the added *L* layer metal film. The script “0” corresponds to no metal layers on the substrate. Here, γ_t_, γ_b_, and γ_i_ are the free energies of top surface, bottom surface, and interface, respectively, and generally speaking, all three energies are functions of metal film thickness.

To assess the thermodynamic stability of a nanofilm, it is also instructive to define a discrete second difference function, *i.e.*, the “stability index” [54,67]

(7) $$\Delta \mu_{L}=\frac{E_{L+1}+E_{L-1}-2E_{L}}{A}=\alpha_{L+1}+\alpha_{L-1}-2\alpha_{L}$$

where *A* is the area of the supercell base face. Note that Δμ*_L_* is independent of the choice of *E_c_*. For Δμ*_L_* < 0, a film with thickness *L* is unstable as it can lower its free energy by bifurcating into films of thickness *L* − 1 and *L* + 1; for Δμ*_L_* ≥ 0, the film is stable against such a bifurcation.

## 3. Pb on Cu(111)

**Figure 2:**
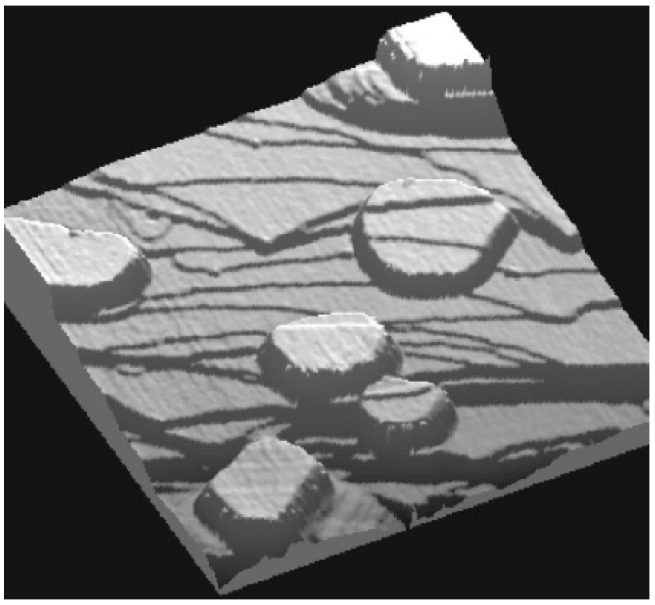
STM image (size: 300 × 300 nm^2^; bias: 1.2 V) showing several Pb islands on a stepped Cu(111) surface. The top surface of the Pb crystallites is atomically flat, but steps on the substrate can still be recognized at the surface of the islands due to lattice-mismatch of Pb and Cu. From [21] (Copyright American Physical Society 2002).

**Figure 3:**
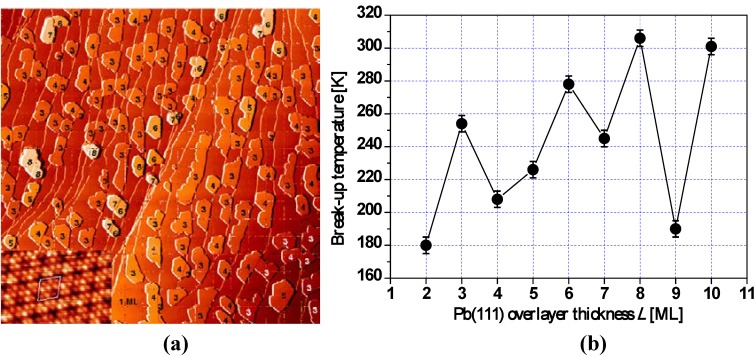
(a) 1000 × 1000 nm^2^ STM image of the morphology of 2 ML of Pb deposited on Cu(111) at 300 K. The inset shows a 7.5 × 3 nm^2^ STM image with atomic resolution of the Pb wetting layer indicating the 4 × 4 reconstruction. (b) Experimentally determined break-up temperatures for different thicknesses of Pb/Cu(111). Adapted from [22] (Copyright Elsevier 2007).

QSE was postulated to explain the apparent “disrupted” bilayer growth mode originally suggested by high-resolution helium atom scattering studies of the low temperature deposition of Pb on Cu(111) at 140 K [71,72]. The height distribution of flat-top Pb(111) nanoislands grown on Cu(111) [at 300 K and annealed to 400 K] has been experimentally obtained from STM analysis by Otero *et al.* [21] Figure 2 provides a sample STM image of such islands. This analysis showed that certain Pb(111) film thicknesses of *L* = 5, 9, 12, 13, 14, 18, and 19 ML are almost forbidden, while *L* = 6, 8, 11, 15, 17, and 20 ML are strongly preferred, the latter thicknesses being “magic”. Dil *et al.* [23] observed the preferred heights at *L* = 6, 8, 10, 15, 18, and 22 ML by angle-resolved photoemission. The discrepancy of thickness stability for *L* = 10 (or 11), and 17 (or 18) ML from the above two experimental groups is attributed to a small change in the boundary conditions at the Pb-Cu interface [23]. More recently, Calleja *et al.* studied the morphology and electronic structure of Pb films grown on Cu(111) using variable temperature STM [22], as shown in Figure 3a. By measuring break-up (bifurcation) temperatures for Pb(111) films with different thicknesses *L* < 11 ML, they determined more stable thicknesses at *L* = 6, 8, and 10 ML than 4, 5, 7, and 9 ML. These results are shown in Figure 3b.

**Figure 4:**
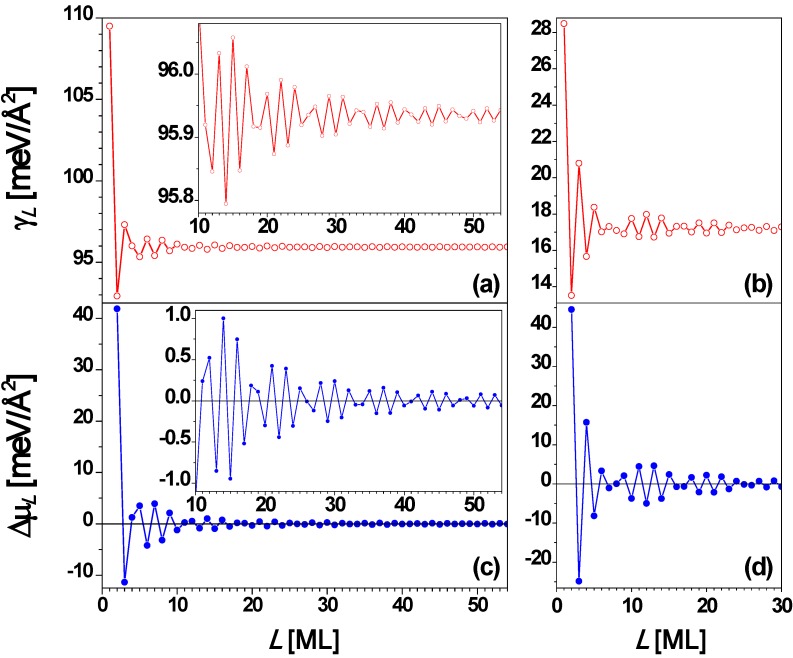
Surface free energy γ*_L_* *versus* thickness *L* for a freestanding Pb(111) nanofilm from (a) EGM and (b) DFT calculations, respectively. Stability index Δμ*_L_* *versus* *L* from (c) EGM and (d) DFT calculations, respectively. The insets are the corresponding local enlargement. From [67] (Copyright American Physical Society 2009).

From the above experimental results, it is clear that the stability of Pb(111) nanofilms exhibits a bilayer alternation disrupted only at 5 layers. To understand the QSE of Pb(111) films supported on a substrate, it is instructive to analyze behavior for *freestanding* Pb(111) nanofilms. This has been done by many groups [26,27,53,67,73]. The interlayer spacing of a Pb(111) film is *d* = 2.8377 Å, and the Fermi wavelength is taken as λ_F_ = 3.9615 Å. Then the smallest integer *j* satisfying Equation (3) is 2 when *m* = 3 (*i.e.*, 2*d* ≈ 3λ_F_/2 in this case) so that the oscillation period is 2 ML. This explains the stability bilayer alternation observed in the above experiments. The surface free energy γ*_L_* and the stability index Δμ*_L_* *versus* nanofilm thickness *L* from the EGM [67] are, respectively, plotted in Figure 4a and Figure 4c showing the oscillation period of 2 ML with a beating pattern of a period of 7.4 ML from Equation (4). Figure 4b and Figure 4d show the plots of γ*_L_* and Δμ*_L_* *versus* *L*, respectively, from the DFT calculations for *L* = 1 to 31 ML. By comparing Figure 4a with Figure 4b as well as Figure 4c with Figure 4d, the oscillation behavior of γ*_L_* and Δμ*_L_* *versus* *L* from the EGM calculations are in overall agreement with the corresponding results from DFT calculations. From Figure 4, the period of the beating pattern is ~ 9 ML. This beating effect can also be observed from experiments. For example, the Pb(111) nanoislands with thicknesses of *L* = 3, 6, 8, and 10 ML are stable at higher temperatures. Below 5 ML, the odd thicknesses (*L* = 1 and 3 ML) are more stable. Note that *L* = 1 ML corresponds to the wetting layer, indicating the Stranski–Krastanov (SK) growth mode [36] of Pb on Cu(111). The curve in Figure 4 shows the existence of the beating pattern with switches in the stable thicknesses between even and odd.

The large overestimate of the magnitude of the surface energy for Pb by the EGM is expected given the high valence and thus electron density for Pb. The electrons are likely over-confined in our EGM treatment. The initial oscillation amplitude for the free energy is somewhat smaller for EGM compared to DFT results, and significantly smaller for thicker films of 10-16 ML. We note the perspective of some workers is that since the Fermi energy lies beyond the first Brillouin zone, one should adopt a rather different effective Fermi wavelength of λ_F_ = 10.3 Å [74]. Such a larger value would induce slower decay than in our EGM (with much smaller λ_F_).

To account for the effect of the substrate, Ogando *et al.* studied the Pb/Cu(111) system via self-consistent electronic structure calculations [24], in which the Cu(111) substrate is modeled with a one-dimensional pseudo-potential, and Pb islands are represented as stabilized jellium overlayers. Overall, this model can reproduce the basic QSE behavior of the Pb overlayer in the above experiments, but there is some mismatch between the model and the experiments. Pb islands of 13 ML are not especially abundant in the above experiments of Otero *et al.*, while islands with this thickness are stable according to the results from the model. The odd-even switch points from the model are at *L* = 3, 10, and 18 ML, *i.e.*, there is no switch point at the experimental value of *L* = 5 ML shown in Figure 3b. These discrepancies were attributed to the experimental situation in which the substrate is not flat but contains steps or terraces [24].

**Figure 5:**
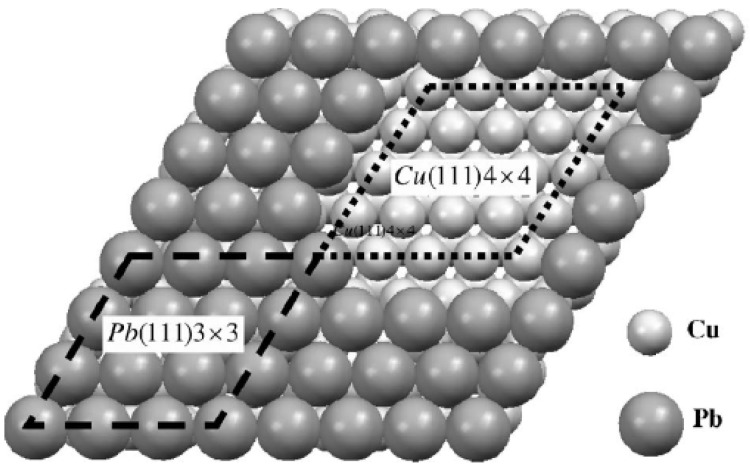
Pb/Cu(111) interface structure adopted in the analysis of showing a match between the 4 × 4 unit cell of Cu(111) and the 3 × 3 unit cell of Pb(111). From [27] (Copyright American Physical Society 2006).

There also exist DFT calculations for supported Pb films on Cu(111) system [26,27]. An obstacle for such calculations is determination of the “real” structure of the Pb-Cu interface. This system is complex, in part due to stain effects resulting from the lattice mismatch between Pb and Cu [26]. It is known that the Pb wetting layer on Cu(111) substrate exhibits a 4 × 4 reconstruction, as shown in the inset of Figure 3b. One simple strategy is to simply strain a freestanding Pb(111) film to match the lattice-constant of the Cu(111) substrate as done by Materzanini *et al.* [26]. Preferably, one can generate a reasonable model for interface structure and perform associated analysis of supported films as done by Jia *et al.* [27]. The choice of interface structure by Jia *et al.* is shown in Figure 5. The basic experimental QSE-related features of film energetics are satisfactorily described by these DFT calculations, e.g., the oscillations with beating effects in energies and work functions [26,27]. Some inconsistency in the stability at certain overlayer thicknesses between DFT and experiment (cf. Figure 3b in Reference [27]) reflects sensitivity to the description of the interface.

## 4. Ag on Fe(100)

**Figure 6:**
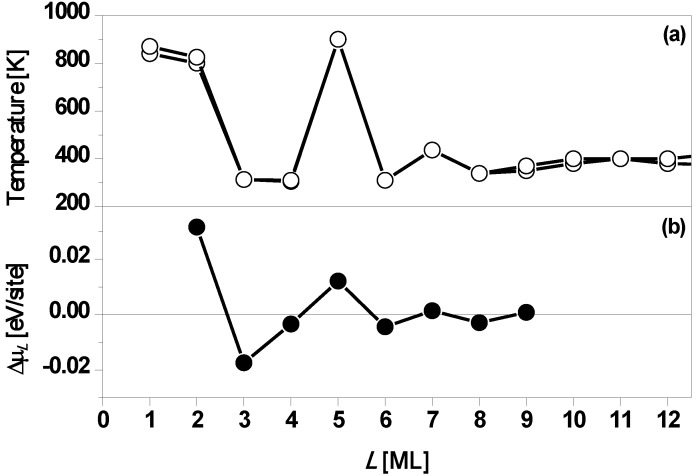
(a) Temperatures at which Ag(100) films with an initial thickness of L on Fe(100) surface begin to bifurcate. The *L* = 5 ML film is the most stable. Multiple data points are shown for several thicknesses as an indication of the degree of reproducibility. (b) Stability index Δμ*_L_* *versus* *L* from DFT calculations. Adapted from [33] (Copyright American Physical Society 2003).

**Figure 7:**
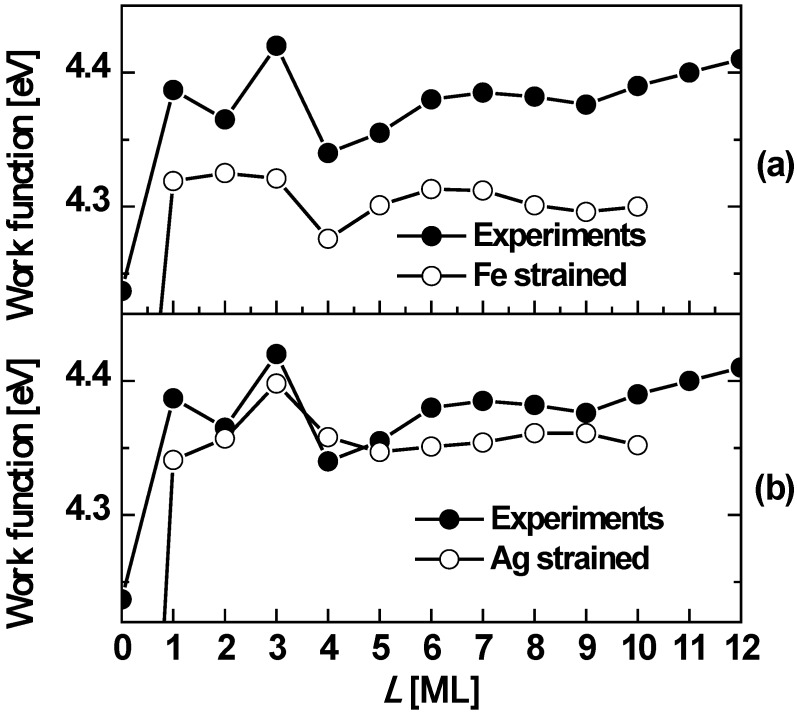
The work function *versus* Ag(100) film thickness on Fe(100). The curves with open circles correspond to DFT calculations: (a) with the theoretical in-plane lattice constant of the Fe substrate is slightly strained to conform to that of Ag; (b) with the theoretical in-plane lattice constant of Ag on a Fe substrate strained to conform to an unstrained Fe substrate (presumably representing the experimental situation for defect free films). In both cases, the (identical) curve with filled circles is the experimentally measured work function. Adapted from [58] (Copyright American Physical Society 2002).

As noted in the introduction, Ag on Fe(100) is an ideal system in which to investigate QSE. The small lateral lattice mismatch (less than 1%) for fcc Ag on bcc Fe in the [100] direction ensures growth of an epitaxial film with a fully-characterizable simple interface, *i.e.*, Ag at four-fold hollow sites on Fe(100) [33]. Chiang *et al.* [29] used photoemission spectroscopy to analyze Ag(100) films of *L* = 1 to 15 ML formed by depositing Ag from an effusion cell onto Fe(100) surface. A key observation was that films of *L* = 1, 2, and 5 ML are structurally stable for temperatures up to around 800 K, whereas films of other thicknesses are unstable and bifurcate into the films with *L* ± 1 ML at temperatures around 400 K. This behavior is shown in Figure 6a. Figure 6b shows the stability index Δμ*_L_* *versus* *L* from DFT calculations of Wei and Chou [33]. From Figure 6b, it is clear that Δμ*_L_* > 0 with large magnitudes for *L* = 2 and 5, implying Ag(100) films of *L* = 2 and 5 ML on Fe(100) surface are particularly stable. Thus, the DFT results are in agreement with the experimental results. Results below for freestanding Ag(100) films clarify the expected influence of the Fe(100) substrate on the stability of Ag/Fe(100) films. In addition to properties related to film stability, it is instructive to also explore the variation with thickness of the work function. Figure 7 shows both experimental and theoretical behavior suggesting oscillations with a period of ~5 ML.

**Figure 8:**
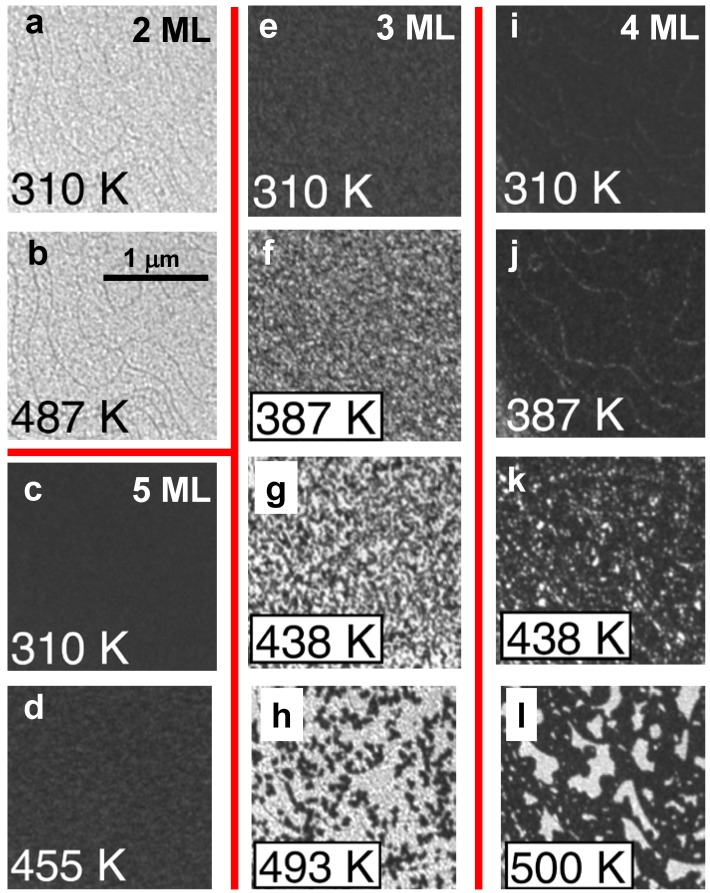
LEEM images of initially [(a) and (b)] 2 ML, [(c) and (d) 5 ML, [(e) to (h)] 3 ML, and [(i) to (l)] 4 ML Ag films at the temperatures indicated during annealing. Adapted from [32] (Copyright American Physical Society 2010).

Evolution of the Ag(100) film morphology on Fe(100) during growth and post-deposition annealing has been monitored by Man *et al.* [31,32] using low energy electron microscopy (LEEM). These investigations also show that Ag films grown at room temperature with thickness *L* = 2 and 5 ML are stable, and that films with *L* = 3 and 4 ML are unstable during annealing. See Figure 8. The films of *L* = 3 and 4 ML were observed to decompose to stable 2 and 5 ML components, instead of undergoing *L* → *L* ± 1 bifurcations. This analysis is described in more detail below

LEEM images for films of different initial thicknesses were recorded at several temperatures while heating the film incrementally up to about 500 K, as shown in Figure 8. Only insignificant changes appear for 2 ML (from Figure 8a to Figure 8b) and 5 ML (from Figure 8c to Figure 8d) indicating that 2 and 5 ML films are stable during annealing. This behavior contrasts with dramatic changes in the images from Figure 8e to Figure 8l. According to Man *et al.*’s analysis, the morphological changes in the 3 ML film are initiated at numerous localized points randomly distributed over the surface (Figure 8f). The morphological transformation nucleates and grows as the temperature is raised. In contrast, the morphological changes in the 4 ML film appear to start at step edges (Figure 8j), although randomly located nuclei of the transformed regions again proliferate and grow as the temperature is raised.

Man *et al.* check the consistency of *I*(*V*) spectra of the stable 2 and 5 ML films and those measured locally in the distinct regions of these thicknesses that are produced by thermal decomposition of 3 and 4 ML films. The also checked the consistency with mass conservation of the area fractions of the 2 and 5 ML regions following decomposition form 3 or 4 ML films. They conclude that 3 and 4 ML films decompose to thicknesses stabilized by QWS. The presence of numerous, small and randomly located pinholes in the initial films provides a kinetic pathway promoting decomposition. In contrast, a distinct kinetically-limited bifurcation mode for thermal decomposition was observed in other experiments with atomically flat films in the absence of pinholes [29]. In either case, for a more detailed characterization of morphological evolution, STM would be valuable.

**Figure 9:**
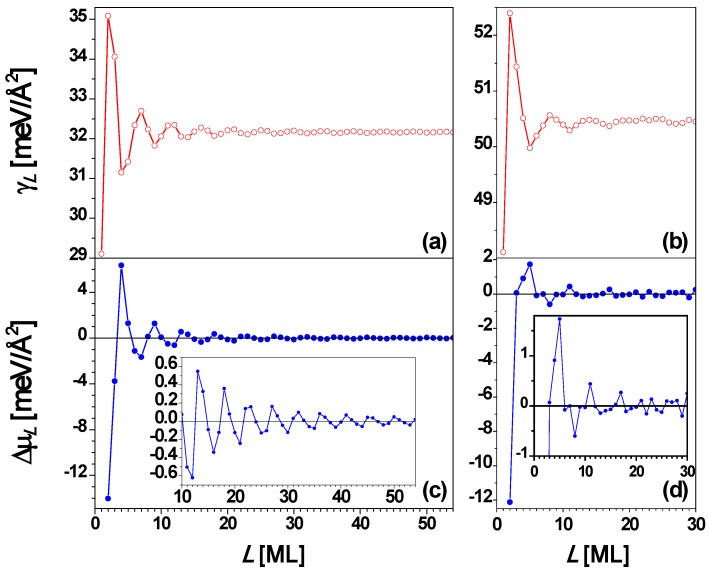
Surface free energy γ*_L_* *versus* thickness *L* for a freestanding Ag(100) nanofilm from (a) EGM and (b) DFT calculations, respectively. Stability index Δμ*_L_* *versus* *L* from (c) EGM and (d) DFT calculations, respectively. The insets are the corresponding local enlargement. From [67] (Copyright American Physical Society 2009).

Finally, for comparison we discuss results from analysis of freestanding Ag(100) films. The Fermi surface of Ag is roughly a sphere, with “necks” along the (111) directions. Thus, for Ag(100) [and Ag(110)] films, QSE can be described well by the EGM [66,67]. For a Ag(100) film, the interlayer spacing *d* = 2.0345 Å, and λ_F_ = 5.2060 Å [67], and then the smallest integer *j* satisfying Equation (3) is 1 when *m* = 1 (*i.e.*, *d* ≈ λ_F_/2 in this case) so that Λ = 4.58 by using Equation (4). Thus, the oscillation period is Λ*d* = 4.58 ML for the Ag(100) film. In Figure 9a and Figure 9c the surface free energy γ*_L_* and the stability index Δμ*_L_* *versus* nanofilm thickness *L* from the EGM [67] are, respectively, plotted, showing the oscillation period of 4.58 ML. Figure 9b and Figure 9d show the plots of γ*_L_* and Δμ*_L_* *versus* *L*, respectively, from the DFT calculations for *L* = 1 to 31 ML [67]. By comparing Figure 9a with Figure 9b as well as Figure 9c with Figure 9d, the curves of γ*_L_* and Δμ*_L_* *versus* *L* from the EGM calculations are in overall agreement with the corresponding results from DFT calculations. Both the EGM and the DFT calculations show that films with *L* = 4 and 5 ML are stable towards bifurcation. The EGM predicts that *L* = 4 ML is more stable, while the DFT predicts that *L* = 5 ML is more stable.

## 5. Cu on fcc Fe on Cu(100)

A general and powerful strategy for creating laterally lattice-matched heteroepitaxial overlayers is to form a coherent strained thin film of element B on substrate A, and then to deposit A on top of the thin layer of B. This strategy has been exploited for the Cu/fcc-Fe/Cu(100) system. QSE in the upper Cu(100) films has also been experimentally demonstrated and analyzed utilizing inverse photoemission (IPE) spectra. In addition, QSE was observed in temperature programmed desorption of carbon monoxide (CO) adsorbed on these upper Cu(100) films of different thickness [74]. In Danese *et al.*’s prototypical system, 5 ML Fe were deposited onto the Cu(100) surface at room temperature. This Fe film grows layer-by-layer in an fcc structure being laterally lattice-matched to the underlying Cu(100) template. The upper Cu overlayer also grows layer-by-layer from 2 to 15 ML thickness. Furthermore, this upper Cu overlayer forms the quantum well in which the electrons are confined. Subsequently, a series of CO adsorption experiments were performed creating CO adlayers chemisorbed on the *L*-monolayers-Cu/fcc-Fe/Cu(100).

**Figure 10:**
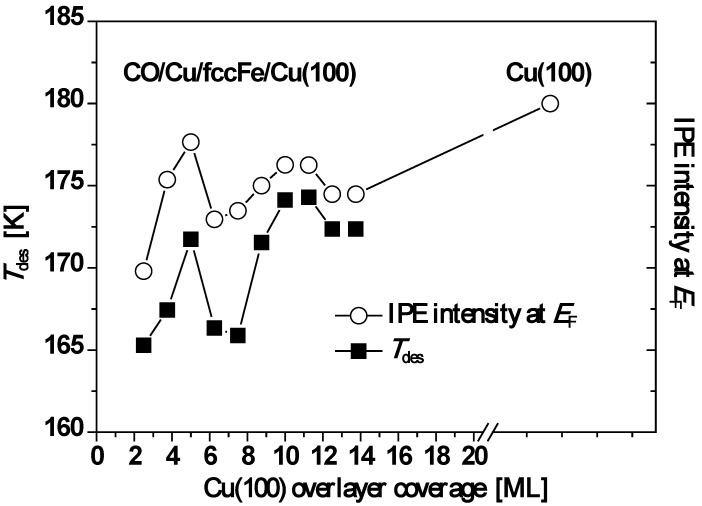
Plot of *T*_des_ (open circles) and *I*(*E*_F_) (closed squares) as a function of Cu thickness. Adapted from [74] (Copyright American Physical Society 2004).

For all film thicknesses, the CO peak desorption temperature, *T*_des_, is lower than that of single-crystal Cu(100). Moreover, oscillations in *T*_des_ are correlated with oscillations in the IPE intensity, *I*(*E*_F_), at the Fermi level, *E*_F_, caused by metallic QWS in the Cu overlayer passing through *E*_F_ as function of film thickness. See Figure 10. Both curves of *T*_des_ and *I*(*E*_F_) in Figure 10 exhibit a local maximum at *L* = 5 ML followed by a minimum near 7.5 ML and then a gradual increase to a second maximum near 10 ML followed by a subsequent decline. For both quantities, the overall trend with increasing Cu thickness is toward the values observed for the single crystal Cu(100) surface.

Our DFT calculations show that for a freestanding Cu(100) thin film, a QWS (with *sp* characteristics) passes through the Fermi level around *L* = 5, and another one passes through between *L* = 11 and 12. This is consistent with the IPE spectra in [75], and we argue that this feature of the QWS is responsible for the oscillations observed in the adsorption energy of CO. The correlation between the CO adsorption energy and the state of *sp* electrons at the Fermi level seems to be reasonable from theoretical calculations showing that CO-metal bonds are dominated by the *sp* electrons on Cu(100) [76,77].

**Figure 11:**
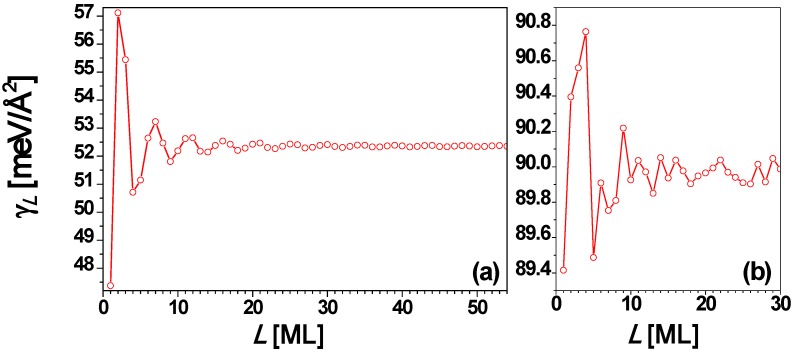
Surface free energy γ*_L_* *versus* thickness *L* for a freestanding Cu(100) nanofilm from (a) EGM and (b) DFT calculations, respectively.

Analogous to Ag(100) films in Section 4, we can analyze the surface free energy of Cu(100) films using both the EGM and DFT calculations. The experimental lattice constant of Cu at 0 K is 3.6024 Å [78]. Then, for a Cu(100) film, the interlayer spacing *d* = 1.8012 Å, and we set λ_F_ = 4.6091 Å. Just as for the case of an Ag(100) film discussed above, the smallest integer *j* satisfying Equation (3) is 1 when *m* = 1 (*i.e.*, *d* ≈ λ_F_/2) so that Λ = 4.6 by using Equation (4). Thus, the oscillation period is Λ*d* = 4.6 ML for the Cu(100) film. In fact, the identical oscillation period for Ag(100) and Cu(100) follows since they have the same Miller index of fcc structure and the same valence number [54]. At least superficially, there is some similarity between the nature of the variation in surface energy for Cu(100) up to ~10 layers and that seen in the experimental *T*_des_ and IEP (cf. Figure 10 and Figure 11). Based on a series of convergence tests for Δμ*_L_* *versus* *L*, we use the **k**-point mesh of 52 × 52 × 1 in the DFT calculations for freestanding Cu(100) films. For more details of the DFT calculations, see [67].

## 6. Ag on NiAl(110)

We now consider the deposition of Ag (fcc structure, lattice constant *a*_Ag_ = 0.409 nm) onto NiAl(110) (CsCl bcc-like structure, lattice constant *a*_NiAl_ = 0.289 nm) [46,57,58]. Despite the difference in crystal structure, there is a virtually perfect in-plane lattice match (*a*_Ag_ = $\sqrt{2}$ *a*_NiAl_) between Ag(110) and NiAl(110) [46,54]. One cannot be certain a priori whether Ag would adopt an fcc(110) structure atop this NiAl template, but this proves to be the case. Thus, this combination of Ag and NiAl(110) provides a simple fully-characterizable epitaxial interface facilitating high-level DFT analysis of supported films. General theoretical considerations (see below) indicate that Ag(110) films should display QSE with strong bilayer oscillations, also characteristic of Pb(111) films. Thus, epitaxial Ag(110) films on NiAl(110) constitutes a system which combines the most appealing and interesting features of the Pb/Cu(111) and Ag/Fe(100) systems discussed in Section 3 and Section 4, respectively.

**Figure 12:**
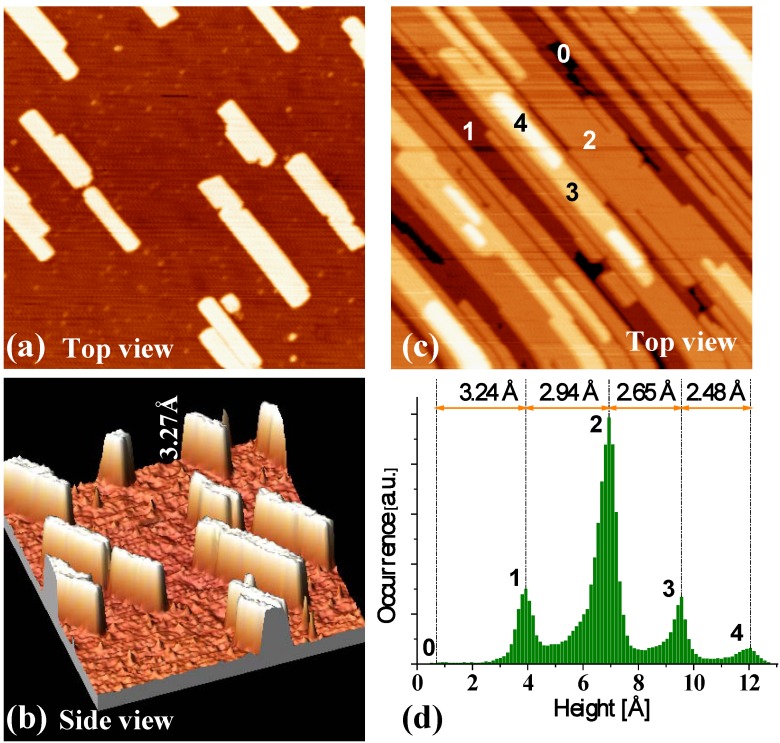
STM images (size: 100 × 100 nm^2^) for Ag deposited on NiAl(110) at 200 K. Flux is 0.0066 ML/s. (a) Top view at Ag coverage of 0.3 ML. (b) A side view of (a). The measured island height is marked. (c) Top view at Ag coverage of 4.0 ML. The island (or film) height levels are labeled. (d) Pixel height histogram corresponding to (c). Here 0 is determined to be the height of the (completely covered) substrate from analysis of height histograms for a sequence of lower coverages. Adapted from [46] (Copyright American Physical Society 2007).

Our experiments for Ag deposition from a Knudsen cell onto NiAl(110) do in fact reveal an initial bilayer-by-bilayer growth mode of the Ag(110) nanofilm (or nanoislands) [46,57,79]. Figure 12a and Figure 12b show the STM images from our experiments for deposition at 200 K and with a low coverage of 0.3 ML. The shapes of Ag islands formed on NiAl(110) surface are rectangular (Figure 12a), and the heights of the islands are measured to be ~3.3 Å (Figure 12b). Figure 12c show an STM image at the higher coverage of 4.0 ML showing the multilayer film morphology. The height of the top of second-level islands is ~6.2 Å measured from the substrate. Figure 12d provides a height histogram corresponding to Figure 12c, and from which the step heights of islands can be determined. For more details and data from the experiments, see Reference [46]. Based on the values of the step heights, a separate LEED analysis, and the results from our DFT calculations described below, we conclude that the Ag islands grown on NiAl(110) surface are of bilayer Ag(110) structure, *i.e.*, the initial growth is bilayer-by-bilayer (at least for the first three bilayers).

**Figure 13:**
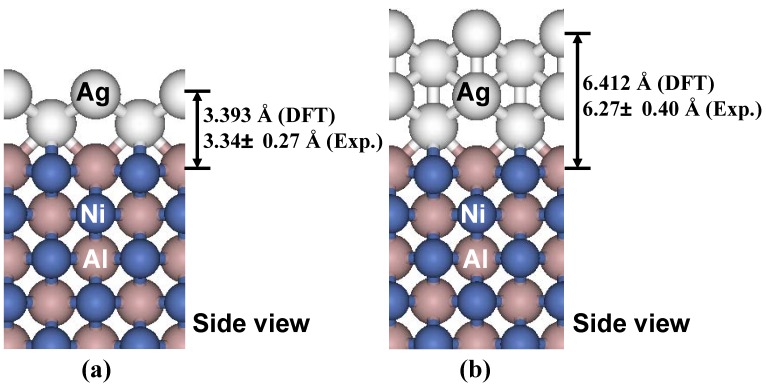
Relaxed structure (side view) of (a) 2 ML Ag(110) adlayer, and (b) 4 ML Ag(110) adlayer on a 11 ML NiAl(110) substrate as determined from the DFT calculations. The theoretical values (DFT) of the Ag(110) adlayer heights and the corresponding experimental values (Exp.) from line-profile analysis [46] are marked.

To determine theoretical values for the interlayer spacing of Ag(110) adlayers on NiAl(110) substrate, a series of DFT calculations with different adlayer thicknesses have been performed [46]. Figure 13 show the relaxed structures for 2 ML and 4 ML Ag(110) adlayers on an 11 ML NiAl(110) substrate. The theoretical values for the heights are in very good agreement with the corresponding experimental values of island step heights obtained from line-profile for islands in levels 1 and 2 (cf. the height histogram in Figure 12d). For more details, see [46,79].

**Figure 14:**
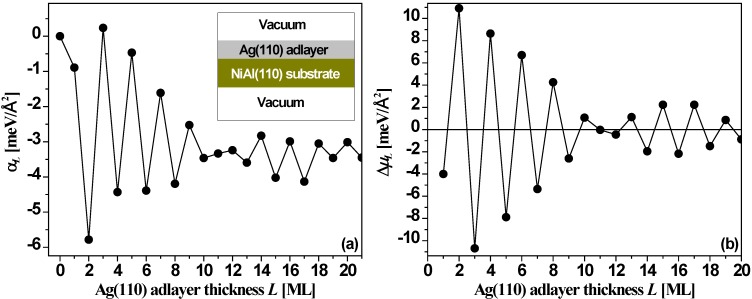
(a) Relative surface free energy α*_L_* from Equation (6), and (b) stability index Δμ*_L_* from Equation (7), *versus* Ag(110) adlayer thickness *L* on NiAl(110) substrate from DFT calculations. Adapted from [54] (Copyright Elsevier 2008).

Figure 14 shows DFT values for the relative surface free energy, α*_L_*, and stability index, Δμ*_L_*, *versus* the thickness *L* of a Ag(110) film supported on an 11-layer NiAl(110) slab. The results exhibit a strong bilayer oscillation pattern again with clear beating. The stable thicknesses for supported films become *L* = 2, 4, 6, 8, 10, and then 11, 13, 15, 17, 19. Other thicknesses are unstable with the odd-even switch points at *L* = 1 (trivial), and 12 (within the first 21 layers). The NiAl(110) substrate plays a role modifying behavior relative to that for the freestanding film (discussed below), stabilizing even rather than odd film thicknesses up to 10 ML. Tests from DFT calculations [54] with different substrate thicknesses all show the oscillation pattern of Figure 14 with only small changes in the absolute values of α*_L_* and Δμ*_L_*.

As discussed above, the STM experiments for Ag deposition on NiAl(110) surface reveal initial bilayer-by-bilayer growth which we attribute to the QSE. It should be mentioned that for thicker films, experimental step heights appear to gradually approach a value similar to that for the Ag(111) interlayer spacing (~2.36 Å). This suggests that the film structure may be transforming from Ag(110) towards Ag(111). Additional experimental evidence is given in Reference [46]. Presumably, this trend at least in part reflects the feature that Ag(111) has a lower surface free energy than other crystalline planes of Ag. Thus, the beating effect discussed below cannot be accessed in these experiments.

**Figure 15:**
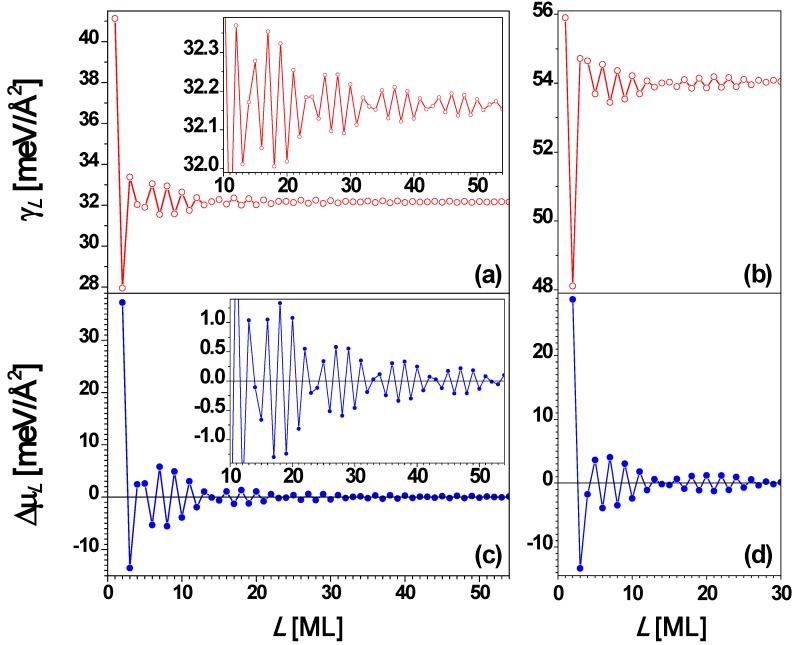
Surface free energy γ*_L_* *versus* freestanding Ag(110) nanofilm thickness *L* from (a) EGM and (b) DFT calculations, respectively. Stability index Δμ*_L_* *versus* *L* from (c) EGM and (d) DFT calculations, respectively. The insets are the corresponding local enlargement. From [67] (Copyright American Physical Society 2009).

Finally, we briefly review EGM and DFT results for the behavior of freestanding Ag(100) films for comparison with the above results for supported films. For Ag(110) film, the interlayer spacing *d* = 1.4386 Å, λ_F_ = 5.2060 Å, and λ_F_/(2*d*) = 1.8094 ≈ 2, so from Equation (3), Λ = 9.49. There should be a primary oscillation with the period of 2 ML and a beating pattern for the envelope of the primary oscillation with a period of ~ 9.5 ML. This is confirmed in Figure 15a and Figure 15c. For the Ag(110) nanofilm, the surface free energy γ*_L_* (Figure 15a) and stability index Δμ*_L_* (Figure 15c) *versus* *L* obtained from the EGM calculations are in good qualitative agreement with the corresponding results (Figure 15b and Figure 15d) from DFT calculations. Note that relative to the NiAl(110)-supported Ag(110) film, there is an apparent shift in the stability pattern toward the smaller *L* direction, *i.e.*, the stability of any thickness *L* in Figure 14 can be obtained from that for *L*–Δ in Figure 15b and Figure 15d. A shift of Δ = 3 seems to work better for smaller *L*’s, while a shift of Δ = 1 seems to work better for larger *L*’s.

## 7. Fe on Cu_3_Au(001)

Both He atom diffraction [80] and photoelectron-diffraction [81] studies have been performed for Fe films on Cu_3_Au(001). The basic observation is that only bilayer islands are formed at 140 K without any surface segregation. After annealing to 400 K, these islands restructure to form mainly trilayer islands. Surface segregation is shown to be inhibited upon deposition at low T and also after the annealing process. It has been suggested that electron confinement might drive QSE-mediated bilayer island formation, prompting our inclusion of this system in the current review. Specifically, Verdini *et al.* speculated that behavior might arise from confinement of the Fe *d* electrons [81]. However, the bilayer growth in this system might not originate from the QSE. For example, we have already mentioned the possibility that kinetic limitations to higher-layer nucleation can produce bilayer islands in SK or VW growth systems. Also, a recent paper provided an analysis of the thermodynamic exclusion of thinner (e.g., monolayer) islands due to stress effects [82].

**Figure 16:**
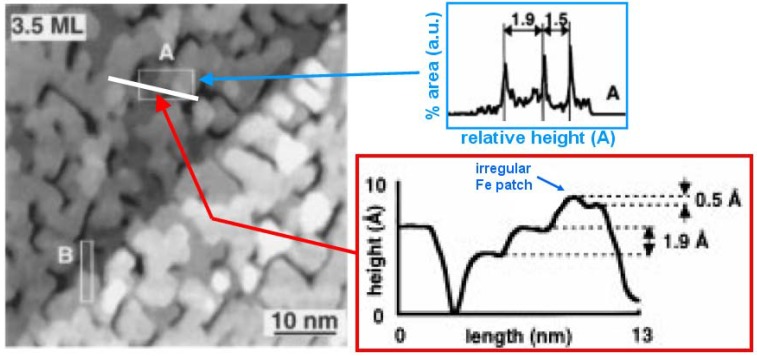
STM image of Fe films grown on Cu_3_Au(001) at 300 K with coverage 3.5 ML. The height distribution plotted in the right hand panel is for area A, as indicated by a white rectangle in the STM image. The area A is particularly chosen to include the regular Fe layers (gray and black) as well as an irregular Fe patch (white gray). This height distribution reveals two different layer distances: 1.9 Å and 1.5 Å, corresponding to the values of the vertical interlayer distances of fcc-like Fe(001) and bcc-like Fe(001), respectively. The lower left panel shows a line profile across this region indicating a bilayer fcc(100) Fe island toped by a third fcc(100) Fe layer, with additional non-fcc(100) Fe on top. A layer distance 3.8 Å (not shown) was obtained from the area B is consistent with the height of a bilayer step on the Cu_3_Au(001) surface separating terraces with the same termination. Adapted from [84] (Copyright Elsevier 1998).

Earlier STM and LEED analysis of Fe films deposited on Cu_3_Au(001) at 160 and 300 K have been performed by Lin *et al.* [83,84]. Multilayer islands are formed for both growth temperatures. In addition, an fcc-to-bcc structural transformation starts at the coverage of ~3.5 and 5.5 ML for the growth temperatures of 300 and 160 K, respectively. This transformation is accompanied by a distinct change in the surface topography. An STM image (Figure 16) shows a typical morphology for 3.5 ML Fe deposited at 300 K. Perhaps of most relevance here is the line profile on the lower right. The groove down to the substrate appears to occur at the edge of a bilayer fcc(100) Fe island which has on top a third fcc(110) layer of Fe (single layer height 1.9 A). On top of the third layer, the higher layer material is perhaps transforming to bcc Fe.

## 8. Ag on 5-Fold i-Al-Pd-Mn and Bi on 5-Fold i-Al-Cu-Fe

Experimental evidence exists that QSE can also affect the morphology of metal thin films grown on Al-rich 5-fold icosohedral quasicrystalline substrates at room temperature or above [85]. These substrates present structural and chemical order of higher complexity than conventional crystalline metal or semiconductor substrates. Al-rich quasicrystals exhibit a deep minimum in the electronic density of states at the Fermi level (*i.e.*, a pseudo-gap) due to both structural and *sp*-*d* hybridization effects. This could induce electron confinement in overlayer films. Based on STM observations, QSE in these systems is proposed to manifest itself in the formation of islands with ‘‘magic height’’, just as for simpler systems. We describe observations made for two different metals deposited on two different quasicrystalline systems, suggesting that the QSE may be quite common for quasicrystalline substrates.

**Figure 17:**
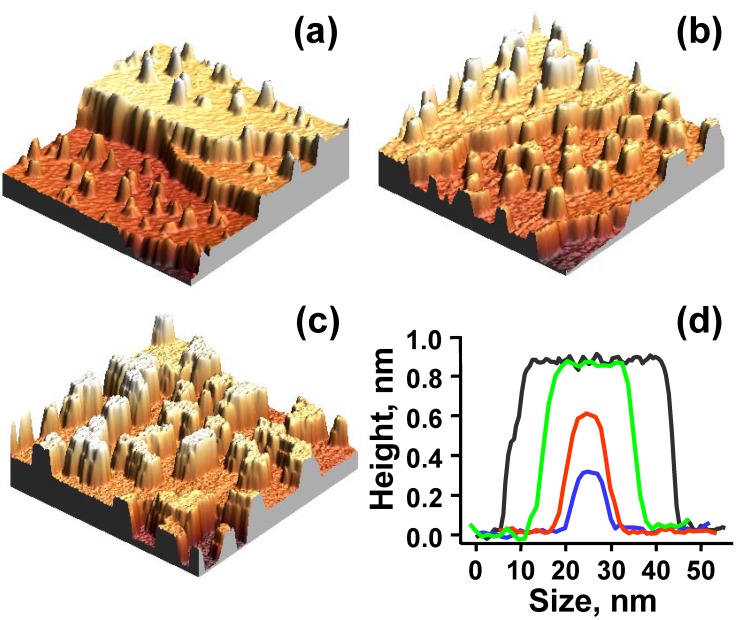
STM images (243 × 243 nm^2^) for Ag films for (a) 0.26 ML, (b) 0.70 ML, and (c) 1.0 ML. (d) Line profiles from typical individual Ag islands showing the sequence of vertical island shape profiles during growth (illustrating height selection followed by lateral spreading). Tunneling conditions: +0.95–0.97 V, 0.44 nA. From [87] (Copyright American Physical Society 2009).

Experimental STM studies of the deposition of Ag on 5-fold i-Al-Pd-Mn at 365 K indicate facile conversion of isolated 2D islands into 3D islands. These 3D islands quickly grow to a “selected height” and then spread laterally [86,87]. In this case, flat-top islands grow directly onto the bare quasiperiodic substrate without formation of a wetting layer. This is Volmer-Weber type growth [36] although impacted by QSE in this system. See Figure 17. Within the quasicrystalline substrate, different “layers” of atoms actually consist of a few vertically-closely-spaced planes of atoms [88,89]. Likewise, the 3D Ag islands presumably consist of such composite layers, where a selected height of 3 layers is observed. However, the height of each layer varies somewhat from island to island on average being 0.26, 0.27, 0.29 nm for layers 1, 2, 3, respectively. The height decreases to 0.24 ± 0.03 nm for layer 4, which is finally populated after significant merging of 3rd layer islands [87]. This suggests that a strongly non-fcc(111) pseudo-quasicrystalline structure for the first 3 layers converts to more fcc(111) like structure for higher layers, noting that Ag(111) has a 0.24 nm step height. As indicated above, height-selection was attributed to QSE in this system. A separate ARPES study has demonstrated the existence of QWS in the Ag film on this quasicrystal surface at least for higher coverages [90].

**Figure 18:**
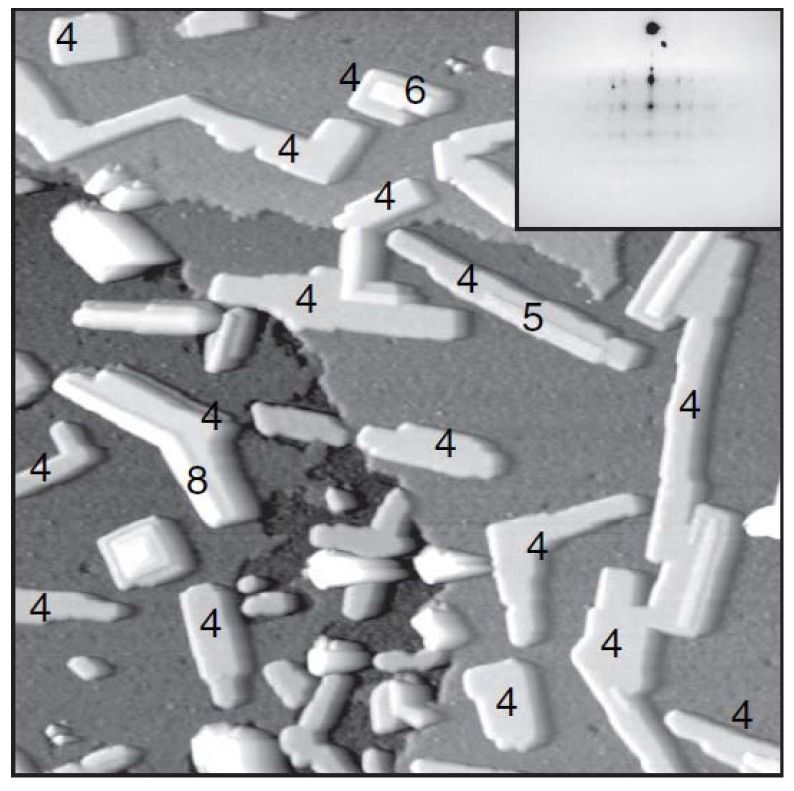
STM topography (400 × 400 nm^2^) of the fivefold Al_63_Cu_24_Fe_13_ surface at the coverage of 4.5 ML Bi. Island heights are indicated in units of Bi(012) ML. The inset shows a typical RHEED pattern observed for the Bi thin film. From [86] (Copyright American Physical Society 2005).

Finally, we describe STM studies of the deposition of Bi on the fivefold surface of the icosoherdal quasicrystal Al_63_Cu_24_Fe_13_ [86,91]. At low coverage (< 1 ML), a Bi wetting layer is first formed, and with increasing coverage, multilayer Bi islands can be observed. This indicates a SK type growth mode [36] although impacted by QSE in this system. Figure 18 shows an STM image at the coverage of 4.5 ML Bi. The distribution of spots form the reflection high-energy electron diffraction (RHEED) pattern in Figure 18 indicates that the flat-top surface of a Bi island is Bi(012) plane. The same RHEED pattern is observed when the sample is rotated *in situ* azimuthally by 2π/5. This implies that the epitaxial relationship within the surface plane is defined by the alignment of a crystallographic axis of the Bi islands with one of five equivalent directions of the Bi wetting layer. Height profiles taken across the Bi islands show that they have a height of 13 Å or a multiple of this value. Because the interlayer spacing of Bi(012) film is 3.28 Å, the height of 13 Å approximately corresponds to a thickness of four Bi(012) ML, or a multiple of 4 ML. In a few cases, 2 ML thick islands could also be observed immediately after deposition, but these islands had irregular shapes and tended to disappear with time, suggesting that such islands are less stable. In any case, 1 ML thick islands were never observed. Thus, the occurrence of a “magic” height of 13 Å reveals a special stability associated with islands of specific thickness.

As an aside, in STM and LEED experiments of Bi deposited on Si(111)-7 × 7 surface by Nagao *et al.* [92], a Bi(012) films of thicknesses 2 and 4 ML above a wetting layer are shown to be stable. When the thickness is larger than 4 ML, the entire bulk volume of the film starts to transform into the Bi(001) phase, as the bulk contribution from cohesion within the film becomes dominant. Based on DFT analysis, the initial bilayer growth is attributed to the pairing of two neighboring layers due to large atomic relaxation to avoid dangling bonds [92,93]. The change from the Bi(012) to the Bi(001) orientation was also observed for Bi growth on the d-Al-Ni-Co substrate (cf. Bi on other quasicrystal substrates [91]).

The valence electrons in Bi are 6*s*^2^6*p*^3^, differing by one electron from 6*s*^2^6*p*^2^ in Pb. We have performed an EGM analysis for the freestanding Bi(012) nanofilm although this analysis should be questioned since Bi is a semi-metal. From the interlayer spacing 3.28 Å of bulk Bi(012) film and the bulk Fermi wavelength of ~3.91 Å, we get a trilayer stability oscillation pattern (not shown) with *L* = 3, 6, 9, 10, 12, 13, … ML stable (here, *L* = 10, 13, … ML are the switch points). This suggests QSE for freestanding Bi(012) films, but the periodicity of oscillation is not consistent with the above experiments of Bi grown on a substrate. Therefore, the physical origin of the magic thicknesses observed for the Bi(012) film in these experiments is still not clear.

## 9. Conclusions

“Electronic growth” in thin films, as manifested by the formation of remarkable height-selected mesa-like quantum islands and films, was discovered via STM studies in 1996 for Ag deposition on GaAs(110) [15]. This sparked tremendous interest in what constituted a novel new film growth mode (in addition to SK and VW growth or to smooth Frank-van der Merwe growth [36]). It is now recognized that this growth mode is a quite general phenomenon which applies not just for metal-on-semiconductor, but also for metal-on-metal systems of the type reviewed here. In addition to Scanning Tunneling Microscopy and Low Energy Electron Microscopy of these height-selected film morphologies, low-energy-electron-diffraction has also provided a valuable tool for the characterization of height selection. Considerable insight into electronic structure which can underlie the thermodynamic preference for this type of growth, *i.e.*, electron confinement and QWS, has been obtained from a variety of experimental methodologies (Photoemission spectroscopy, ARPES, IPE, work function analysis, temperature programmed spectroscopy, *etc*.)

Theoretical analyses have proved a valuable supplement to the experimental studies. Ideally, one would prefer high-level *ab initio* DFT analysis of the electronic structure and properties of supported films. However, a typical impediment to such analyses is a lack of understanding or characterization of the interface between the film and substrate. Two important exceptions, described in this review, are Ag/Fe(100) and Ag/NiAl(110) where the is a very good lateral lattice-match between the substrate and the appropriate surface of fcc Ag. Lacking characterization of the interface, DFT analysis of unsupported freestanding films has some value, e.g., in determining periodicity of any oscillations in surface properties. Furthermore, for elucidation of such basic behavior, much simpler electron gas models (EGM) have been quite successful and instructive.

A broader and significantly more challenging goal, not discussed in this review, is to provide a detailed elucidation of the growth kinetics in these fascinating heteroepitaxial metal systems. In general QSE are quite weak, so it is perhaps surprising that height-selection is achieved during non-equilibrium growth. Also, these height-selected states are sometimes metastable. Indeed, in some systems, the deposition protocol has to be appropriately selected (often lower deposition temperatures) to achieve height-selection. However, for formation of bilayer or taller islands, there is a need for upward mass transport which is generally kinetically limited at lower *T*. From these observations, one can anticipate the challenges in obtaining a complete understanding of kinetics. However, some recent significant progress has been made in this endeavor [57,87].
